# Internal and External Dynamics in Language: Evidence from Verb Regularity in a Historical Corpus of English

**DOI:** 10.1371/journal.pone.0102882

**Published:** 2014-08-01

**Authors:** Christine F. Cuskley, Martina Pugliese, Claudio Castellano, Francesca Colaiori, Vittorio Loreto, Francesca Tria

**Affiliations:** 1 Institute for Complex Systems (ISC-CNR), Roma, Italy; 2 Sapienza University of Rome, Physics Department, Roma, Italy; 3 Institute for Scientific Interchange (ISI), Torino, Italy; University of Maribor, Slovenia

## Abstract

Human languages are rule governed, but almost invariably these rules have exceptions in the form of irregularities. Since rules in language are efficient and productive, the persistence of irregularity is an anomaly. How does irregularity linger in the face of internal (endogenous) and external (exogenous) pressures to conform to a rule? Here we address this problem by taking a detailed look at simple past tense verbs in the Corpus of Historical American English. The data show that the language is open, with many new verbs entering. At the same time, existing verbs might tend to regularize or irregularize as a consequence of internal dynamics, but overall, the amount of irregularity sustained by the language stays roughly constant over time. Despite continuous vocabulary growth, and presumably, an attendant increase in expressive power, there is no corresponding growth in irregularity. We analyze the set of irregulars, showing they may adhere to a set of minority rules, allowing for increased stability of irregularity over time. These findings contribute to the debate on how language systems become rule governed, and how and why they sustain exceptions to rules, providing insight into the interplay between the emergence and maintenance of rules and exceptions in language.

## Introduction

Language is a continuously evolving system, subject to constant pressures to enhance expressivity while guaranteeing successful communication over time. William D. Whitney [1], one of the earliest American English lexicographers, highlighted that language can be seen as a open system subject both to *conservative* and *alterative* forces. In this view, language is a living system continuously experiencing birth, growth, decay, and death. Accordingly, language changes as a result of external or exogenous pressures, but is also subject to internal or endogenous pressures.

Endogenous factors are related to inner instabilities of languages leading to changes independent of external disturbances: for instance, in phonology, pressure against ambiguity makes sounds maximally contrastive to decrease the likelihood of misunderstanding [2], [3]. More generally, any mistakes or unconscious innovations introduced by speakers could trigger endogenous change in language. On the other hand, exogenous factors are related to historical, political, social, and technological factors that affect language communities [4]–[6]. For instance, an exogenous influx of non-native speakers into a language has been correlated with decreased morphological complexity [7].

Despite a huge literature concerning language change and its causes (e.g., [8], [9]), change is often considered in terms of *either* exogenous *or* endogenous pressures (although see [10]), and quantitative accounts of their combined effects have only recently begun to emerge [11], [12]. The recent availability of large-scale historical corpora, and the use of a more quantitative complex systems approach, has allowed ever increasing detail in documenting language change, from the trajectories of individual words [13], [14] to the broad statistical properties of language over time [15], [16].

Here we leverage this opportunity by examining how endogenous and exogenous pressures affect the interplay between regularity and irregularity in language. To this end, we study the formation of the simple past tense in American English, contrasting regular forms (e.g., *look-looked*) with irregular constructions (e.g., *ring-rang*). The past tense has been considered extensively as a general exemplar for how language is cognitively structured [17], and also as an illustration of how high frequency linguistic variants exhibit greater stability [18]–[20]. Here we take a diachronic perspective, monitoring how regular and irregular forms emerge, stabilize, and change over time. The Corpus of Historical American English (CoHA [21]) is composed with a balanced variety of written genres, including more than 330 million words from the 1830–1989 period, each tagged for part of speech.

CoHA allows us to define regularity in terms of actual usage, giving a proportion of irregular usage for each verb at any given time. We observe variations in regularity of clear exogenous or endogenous nature, coming both from birth (exogenous) and regularization/irregularization (endogenous) processes. We highlight how exogenous and endogenous processes have different impacts on the dynamics of regularity. The expansion of the vocabulary drives the increase in regularity through the entrance of new, mostly regular, verbs. However, at odds with previous findings from historical corpora [18], the endogenous process of regularization does not foster significant changes in overall regularity. Endogenous regularization processes, in fact, turn out to be paralleled by comparable irregularization processes, leaving the number of irregular verbs roughly constant over time.

All this implies that the relationship between verb instability and frequency is more complex than previous corpus studies have indicated [18]. In particular, we show that sound similarity plays a crucial role in the persistence of irregularity. By defining classes of irregular verbs based on sound similarity, the relationship between irregularity and frequency is made clearer. We show that the trajectories of individual verbs and classes over time allow insights into the underlying rule set, informing the longstanding debate regarding whether language is strictly rule-governed with a static set of exceptions [22], or a set of connectionist processes leading to a system that appears to have explicit rules [23], [24]. Rather than either of these extremes, the past tense is comprised of competing set of rules, with the regular rule dominating the majority of verb types, and a collection of minority rules applying to irregular verbs [25]. Our findings show that regularities and exceptions emerge from the dynamic interplay of exogenous and endogenous forces: a push and pull between the external influx of new verbs and a struggle among existing verbs to hit the moving target of a dynamic rule set.

## Results

Our analysis aims at presenting a quantitative and balanced picture of factors contributing to language change. Focusing on verbs as our testing ground, we document the effects of verb birth and death, as well as the transformation of existing words (regularization/irregularization). In order to analyze the effect of the different factors we separate our analysis in two parts. First, we focus on the full dataset spanning 16 decades (1830–1989) in order to characterize the temporal dynamics of an open language system. In this case, the ensemble of verbs form an *extended vocabulary* whose size and composition vary due to verbs entering and exiting the language (data available in file S2). Secondly, we focus on changes occurring in the *core vocabulary* considering only verbs which occur in each of the 16 decades considered. We refer the reader to the Materials and Methods section and the Supporting Information for details on corpus preparation (S3 in file S1) and data analysis (S4 in file S1).

### Language as an open system

**Figure 1:**
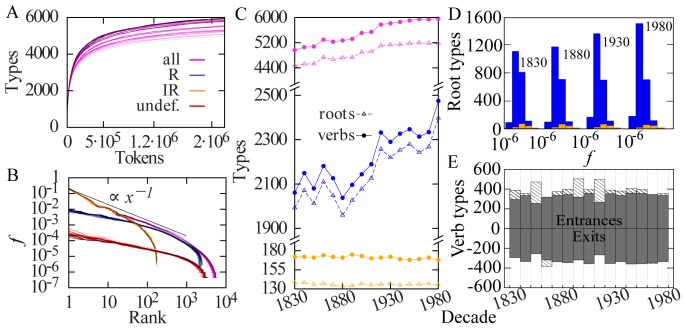
Language as an open system. Colors indicate the type of verb or root depicted: all verbs (purple), mostly regular (blue), mostly irregular (orange), and undefined (red). Lighter saturation of a colour indicates earlier decades, while darker saturation indicates later decades. Where relevant, decades are labelled with the first year, e.g., 1830–1839 is 1830. (A) The number of types versus the number of tokens for all verbs in each decade. Given an equal number of tokens, the increase in the number of types over time reflects a genuine increase in the number of verbs at fixed sample size. (B) Frequency-rank plot contrasting different kinds of verbs (all, mostly regular, mostly irregular, and undefined). Mostly irregular types constitute the highest frequency regime, undefined types constitute the lowest frequency regime. (C) Plot of the number of mostly regular types, mostly irregular types, and all types over time, both for verbs and roots. The number of mostly regular verb types or root types increases over time, while the number of mostly irregular verb and root types remains essentially constant. The sum of all types considered together is more than the sum of mostly regular and irregular types; this is due to this category encompassing undefined types. (D) Frequency distribution of mostly regular, mostly irregular, and undefined categories for root types in four decades, the starting point of the first frequency bin is indicated for each depicted decade. (E) Verb type birth and death by decade. Each bar refers to the entering (top) and exiting (bottom) number of verb types between two decades; lighter areas show the entrance/exit differential. E.g., the first bar depicts the exits after 1830–1839 and the entrances in 1840–1849. There are generally more verbs entering than exiting, indicative of the overall growth in the number of types.

The extent to which language can be seen as an open system can be quantified by looking at the growth in the number of distinct verbs (*types*) as a function of the total occurrences of verbs (*tokens*), the equivalent of Heaps' law [26]. Here, in order to highlight the change in language composition over time, we focus on the different decades separately, considering the same number of verb tokens in each decade (in order to consider genuine growth, rather than increased availability of material for digitized corpora over time; see S3 and Figure S2 in file S1 for further detail). In each decade, types show a sublinear growth as a function of tokens (Fig. 1A), implying that the rate at which new verb types enter the vocabulary decreases with the corpus size. The Heaps' curves for different decades do not overlap; rather, they show a clear increase in the number of verb types in consecutive decades, even with a fixed number of tokens (for full curves, see Figure S1 in file S1; for removal information see Table S1 in file S1). This growth indicates a genuine influx of new verbs entering the language in the time period. Figure S1 (file S1) shows the number of tokens prior and after confinement in each decade.

In order to examine changes in the verb system it is convenient to give an operational definition of regular and irregular verbs. To this end, for each verb and in each decade, we consider the the proportion of irregularity (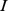) as the number of irregular past tense (non *-ed*) tokens divided by the total number of past tense tokens. For each decade, we count types which are mostly regular (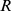) (or mostly irregular, 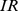) as those which have 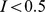 (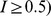. We also provide separate counts for verb types and root types, the latter combining verbs with the same root to level the effects of individual verb productivity (e.g., *do* and *undo* are separate verb types, but instances of a single root type; in file S1, see S2, Table S2, Table S3). Many types do not appear with sufficient frequency to be definitively classified as regular or irregular; such types are categorized as *undefined* (i.e., appear with zero or extremely low frequency of usage in the past tense; in file S1, see S2, Table S2, Table S3). Figure S3 (in file S1) shows, for each category of root types, the histogram in frequency in four different decades (data for root types is available in file S3). Fig. 1B complements the Heaps-like plot by showing frequency-rank plots [27] for different verb types in all decades, showing that the high frequency regime is dominated by irregulars. We now turn to the dynamic properties of the corpus, by explicitly showing how the total number of types grows over time. Fig. 1C shows the number of verb types and root types across decades, for mostly regulars, mostly irregulars, and all types. While there is no significant variation in the number of mostly irregular types, mostly regular types display a clear increasing trend, indicating that entering types are predominantly regular.

The activity and growth of the vocabulary is even more evident in Fig. 1E, where the number of verb types entering or exiting the language in each decade is plotted. The imbalance between entrances and exits (lighter areas) gives rise to the overall growth of the number of verbs over time. Furthermore, Fig. 1D shows the frequency distribution of different kinds of root types, indicating that the growth in the number of types is most prominent for regulars. We can thus conclude that the increase in regularity is driven by an exogenous process of regular verbs entering the language, making for an increase in the ratio of regular types to all verb types as the language evolves.

### Dynamics of the core vocabulary

We now turn our attention toward the endogenous processes of regularization and irregularization. To this aim, we restrict ourselves to types which occur at least once in each of the 16 decades. In addition, in order to capture the genuine dynamics of regularization/irregularization (rather than word formation processes), in the core vocabulary we consider exclusively root types. We thus define the core vocabulary as the set of all (3596) root types which occur at least once in each of the 16 decades. For the present analysis, root types are defined as regular (irregular) in a given time period if their proportion of irregularity 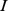 is equal to 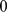 (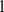) within a tolerance threshold, 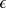 (see S4, file S1). Thus we can further identify *stable regulars* (*stable irregulars*) if they are regular (irregular) in all decades. All other core roots, which have 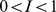 in at least one decade, are considered *active*. This latter set includes roots for which some regularization or irregularization process is in progress, or roots whose regular past tense form coexists with an irregular form. Note that although core roots are by definition present in each decade with some overall frequency, some root types have zero or few occurrences in the past tense, so that their regularity can be undefined in some decades (see file S1: S4, Table S2 and Table S3 for further detail).

The dynamics observed in the core vocabulary are the result of endogenous regularization/irregularization processes only. The first observation is that the vast majority of types are stable regulars or irregulars (types which are regular or irregular in every decade, respectively), and only exhibit fluctuations in frequency (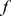). In the midst of these two classes of stable root types, there is a minority of 81 active roots, whose 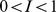 in at least one of the sixteen decades considered.

**Figure 2:**
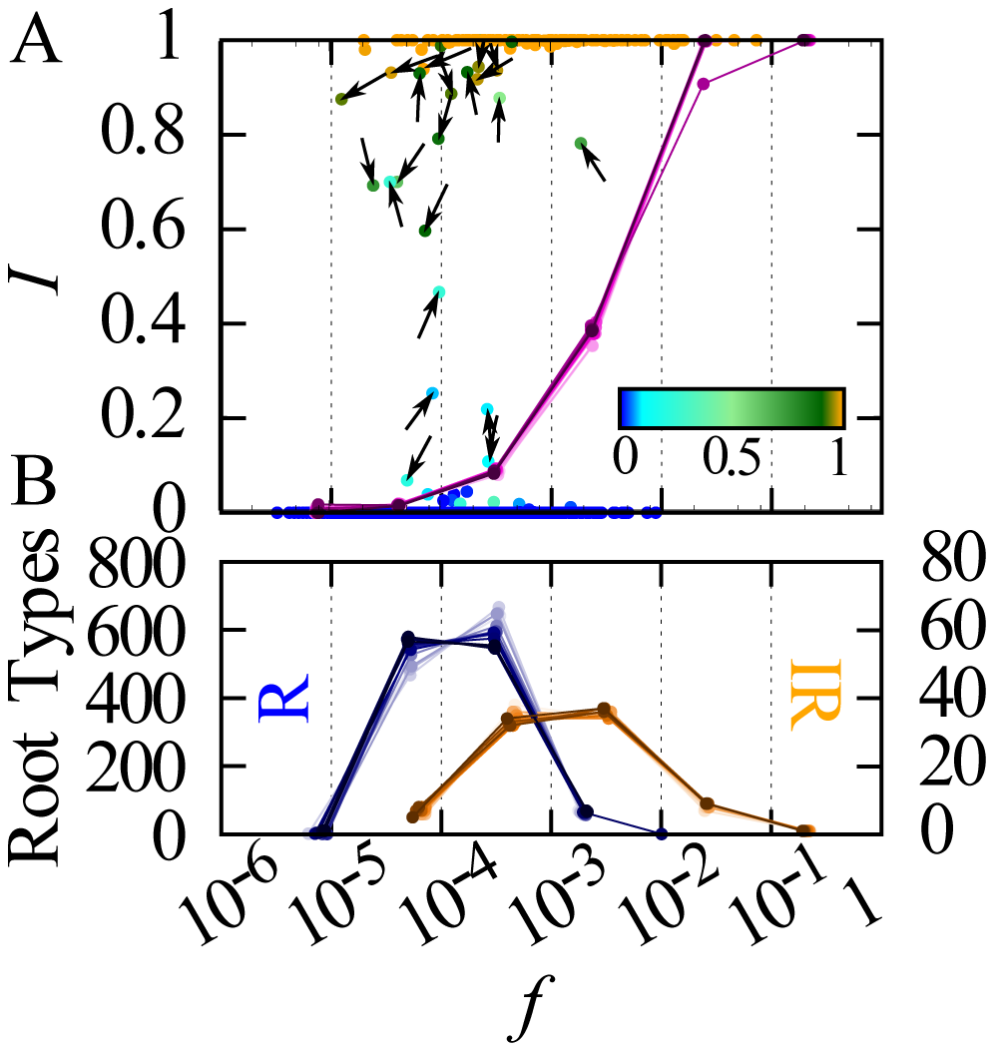
Relationship between frequency 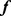 and regularity in the core vocabulary. Lighter saturation of a color indicates earlier decades, while darker saturation indicates later decades. (A) Single points represent roots in the final decade (1980–1989), color-coded according to the average value of 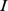 across all 16 decades. Arrows are placed on 21 active verbs with 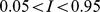. The direction of each arrow indicates the trajectory from the first to the last defined occurrence in the (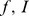) space; the length of arrows is fixed. The purple curves show the binning of both 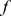 and 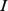 in each frequency logarithmic bin, for each decade. (B) Distributions of the number of stable regular roots (left axis) and stable irregular roots (right axis) for each decade, showing separate peaks which indicate different frequency profiles.

Fig. 2A shows 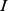 as a function of 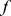 for each of the core roots, considering these values in the last decade (1980–1989) plus the binned values in each decade, which demonstrate that the observed behavior is qualitatively similar across all the decades considered. As already stressed, the overwhelming majority of verbs have 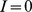 or 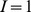, and as other studies have pointed out [18], [28], irregular roots are generally higher in frequency than regular roots. Almost all active root types span a frequency interval between 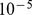 and 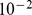. Above this range mainly high frequency irregular roots are present, while below this range predominantly regular roots are observed. The arrows on the active roots indicate the trajectory of the root in the (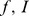) plane from the first decade where the root was defined (i.e., an arrow pointing up indicates an increase in 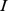 since the first decade, an arrow pointing right indicates an increase in 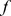 since the first decade, and so on). The observed patterns remain remarkably constant over time, dominated by the stable regulars and irregulars. As expected, the binned value of 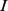 turns out to be a growing function of frequency 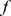, passing from approximately 0 for 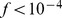 to approximately 1 for 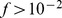. However, the transition from regulars to irregulars is shifted towards high frequencies with respect to the frequency band where we mainly observe active types. This is due to the larger overall number of regular types dominating the irregularity proportion in each bin. Fig. 2B shows that the frequency distributions of stable regular and stable irregular root types are well separated.

**Figure 3:**
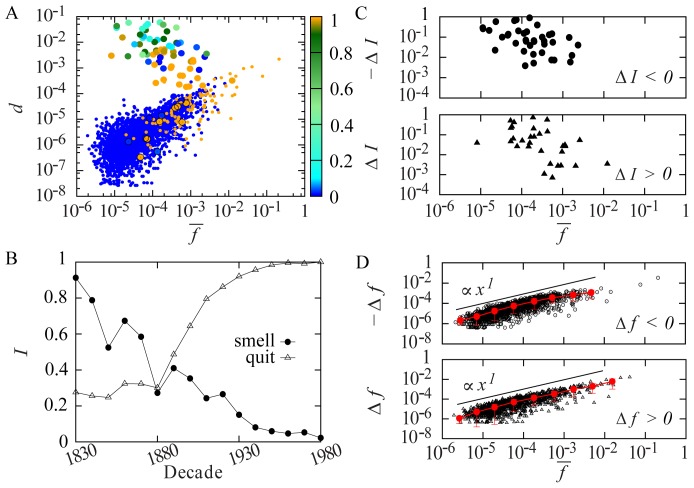
Excursions in frequency and proportion of irregularity in the core vocabulary. (A) Plot of 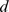 against 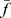 (frequency averaged across all the decades) for core roots. Points are color-coded according to their average 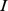 across all the decades. Active roots are represented by larger points, patterning predominantly in the upper “cloud,” distinctly away from stable regular and irregular roots. Some active roots fall into the lower “cloud” (indicated by black circles), due to an effective net 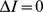 (i.e., 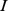 fluctuates below 1 or above 0 between the first and last decade, but has the same value in the first and last decade). (B) Plot of the time evolution of 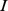 for exemplars of irregularization (*quit*) and regularization (*smell*). (C) Excursion in irregularity proportion 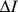 (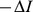 when negative) calculated as the difference in 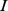 between the first and last decade plotted against 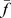 for the 69 active roots with a non-zero 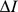 between the first and last occurrences. A negative 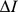 indicates regularization, a positive 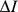 indicates irregularization. The decreasing trend with increasing average frequency corresponds to the behaviour of the “cloud” in (A). (D) Excursion in frequency 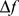 (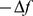 when negative) against 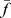 for all core roots. Red points represents binning of 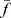 and 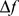, error bars are given for 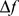. The trend of 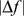 proportional to 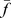 is visible.

The dynamics of roots in the core vocabulary are more deeply understood by measuring the excursion of each root type in the 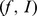 plane, defined as 
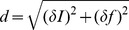
, where 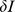 and 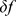 represent the variations of 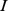 and 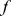 divided by the number of decades where the root is defined (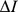 and 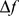 refer to the total change). Fig. 3A shows 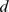 as a function of 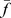, the average frequency across all the decades, for all core root types; points are coloured according to the average 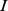 over time. Interestingly, points are clustered in two different regions, a signature of the emergence of two distinct behaviors. Active types are concentrated in the upper cloud, characterized by a decreasing trend of 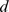 in frequency. Here the variation of 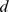 is mostly due to variation of 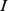 (see also Fig. 3C), although 12 of the active verbs have a 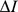 of 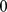 between the first and last decade (though by definition, they have some fluctuation in the intervening decades). On the other hand, stable regulars and irregulars have 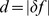 roughly proportional to 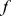 (see also Fig. 3D). In order to disentangle the contributions of frequency and irregularity to 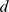, in Fig. 3C and 3D we plot 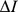 and 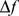 separately as a function of 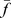; 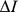 for active roots only (3C) and 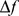 for all core roots (3D).

Interestingly, both the 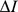 and 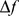 curves appear roughly symmetric with respect to positive and negative variations. As for irregularity variations, the number of verbs changing significantly their 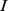 value, and the amplitude of these variations, are roughly the same for regularization and irregularization, indicating a balance in the two processes. Moreover, the changes in 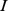 appear to be similar in both directions (towards regularization and towards irregularization) for any fixed value of 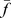, again pointing to the presence of opposite endogenous forces of comparable strength. A similar situation is observed for frequency variations. In particular, while the amplitude of frequency changes seems to be strongly correlated with frequency itself, the direction of the variation appears independent from it, and roughly the same number of verbs increase and decrease their frequency at any given value of 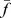. Overall, six roots exhibit dramatic changes in 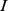 over the 16 decades, with three verbs fully regularizing and three fully irregularizing: *smelled, spilled* and *spelled* overtake *smelt, spilt* and *spelt*, while *quitted, lighted* and *wedded* are replaced by *quit, lit* and *wed*. Fig. 3B shows the proportion of irregular usage 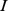 of *quit* and *smell* over time, showing the complete irregularization of *quit* and the regularization of *smell* (see Figure S5, file S1 for the other verbs).

### Relevance of exogenous and endogenous factors

Contrasting the extended vocabulary with the core vocabulary highlights two different types of dynamics in the language. The considerable expansion of the extended vocabulary – new verbs entering the language – is clearly an exogenous process. The dynamics of root types in the core vocabulary exemplify language internal, endogenous processes. Both processes have the potential to affect regularity; however, exogenous dynamics are far more influential in this regard.

The evidence presented so far clearly indicates that overall, the set of verbs tends to become more regular over time: the ratio of mostly irregular root types to mostly regular root types (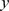) has decreased from 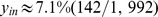 in 1830–1839 to 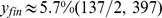 in 1980–1989. This is due to the considerable growth in the number of regulars, while the number of irregulars remains essentially constant throughout 160 years. The contribution of endogenous regularization of existing verbs to the growth in regularity is minimal: the driver of the global regularization process is instead the exogenous birth of new regular types.

The exogenous effect of new regular verbs entering the language is quantitatively overwhelming with respect to that of endogenous regularization. However, while regularity increases in terms of types, it remains constant at the system level. In other words, the total proportion of irregular usage 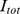 is fairly constant over time: between 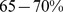 of all past tense verb tokens are irregular (see Figure S6, file S1). Although many new verbs enter the system as regulars, they do so at very low frequency, so that their impact on 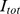 is exceedingly small. High-frequency types, which constitute the bulk of verb usage, tend to be irregular and remain so over time.

### Drivers of regularization and irregularization

Although our data show that the core of the vocabulary is stable overall, there remains a minority of active, dynamic verbs within the language, undergoing processes of regularization or irregularization. Here, we consider what causes this activity from an endogenous perspective.

A good deal of consideration has been given to why regularization occurs. From a system perspective, there may be pressure for individual verbs to conform to the dominant rule [17], creating a system with a more concise overall rule-set attractive to learners [29]. From a learner's perspective, regularization occurs as an individual fails to accurately retrieve an irregular form, and falls back on the dominant regular rule [30]. The phenomenon of irregularization has been given less consideration, as it is often considered so rare as to be irrelevant [18], [31]. However, our data show that in terms of types, irregularization is roughly as prevalent a process as regularization; but what cognitive and system pressures might account for it?

Phonological analogy may be a powerful process in this regard [32], [33]. Although some of the most frequent irregulars are suppletive (i.e., there is little connection between the present and past form, e.g., *be/was*), many irregulars form the past by application of systematic changes to their roots, and share striking similarities with other irregulars (e.g., *sing, ring* 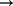 *sang, rang*) [24]. In other words, highly frequent irregular verbs may form attractors, which have the potential to draw phonologically similar regulars. This process could account not only for processes of irregularization, but also for the presence of some anomalous stable irregulars with relatively low frequency. Regularity, on the other hand, is a rule which applies at the morphological level, and thus, regulars do not act as strong attractors for other phonologically similar verbs (however, the regular rule does exhibit phonologically conditioned variation, as in the difference in the spoken realization of *-ed* in *walked* vs. *faded*; [30]). Rather, regularization occurs as a consequence of broad application of a morphological rule, which is applied indiscriminately when an irregular exception is unknown, and this becomes more likely as frequency declines.

To operationalize this perspective, we propose a clustering of the set of irregular root types into classes based on phonological similarity. We group all roots which have some 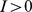 into classes based upon the type of change which occurs to the infinitive to form the past tense. For example, *ring* transforms to *rang*, placing it in the same class as *sing* (see Table S4, file S1 for full listing of classes). Each class has the potential to draw new irregular members over time, but may also lose low frequency members to regularization (see S5, file S1).

**Figure 4:**
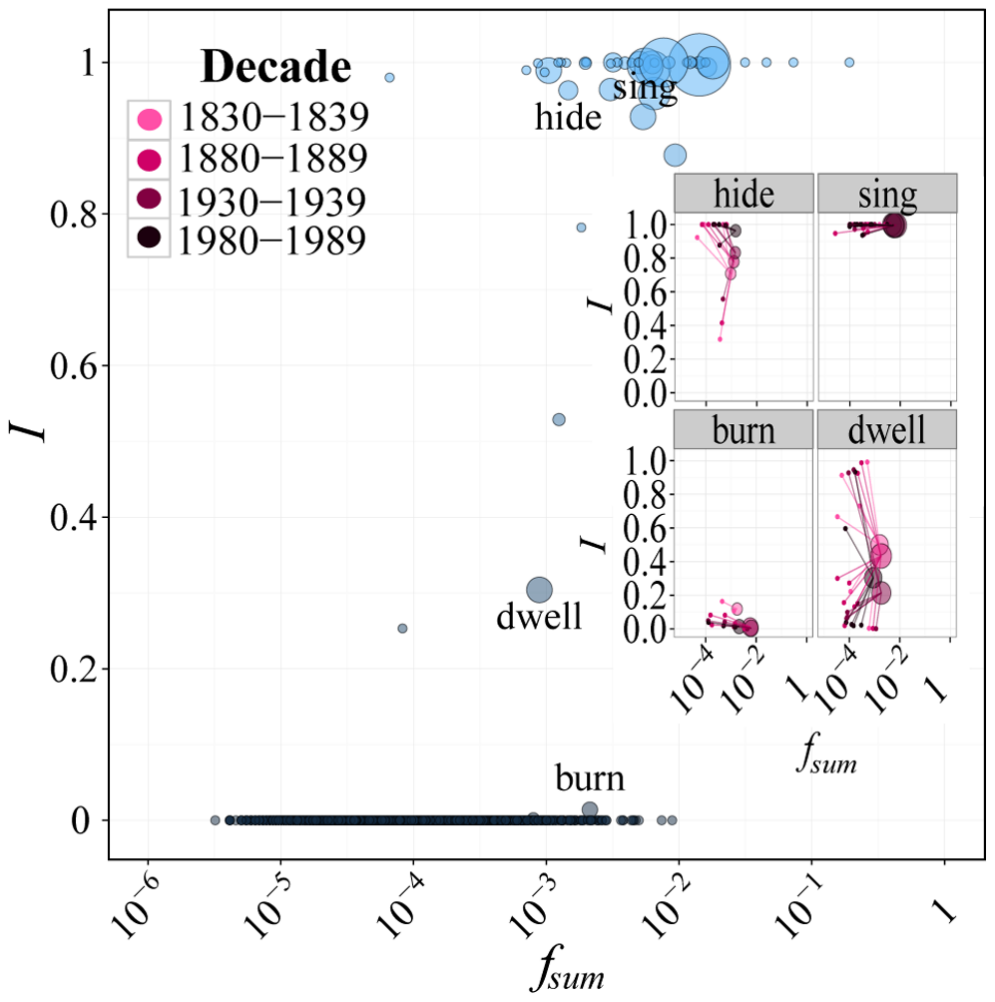
Plot of the proportion of irregularity, 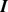, for phonological classes against 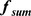, the sumed frequencies of members in a class. The main plot refers to the last decade (1980–1989) and, along with classes, shows regular roots in the decade (grey points at the bottom). For each unclassed regular root, the same values of 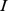 and 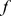 are used as in Fig. 2A. The size of the circle for each class is proportional to the number of members in the class. The insets show four exemplars of classes with different behaviours, in four time snapshots (identified with a purple hue going from light to dark with increasing time): the largely irregular *hide* and *sing* classes on the top, and the regularizing *burn* and *dwell* classes on the bottom. Small points in the insets are the member roots of the class (with their values of 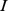 and 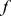), connected with lines to the class itself. The relationship between variance of 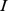 within a class and its stability is visible especially in the “star-like” quality of the *dwell* class. The plot shows that even basic phonological classification makes the frequency/regularity relationship clearer, as compared to Fig. 2A.

Nothing guarantees that this criterion provides an optimal classification, and the quest for irregular classes which align most closely with the actual grammars of speakers is a challenging open problem. However, even this basic classification has the effect of clarifying the otherwise anomalous behavior of some verbs, and making the separation in frequency between regulars and irregulars more distinct by shifting lower frequency irregulars. Fig. 4 displays 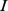 for a given class (calculated as all non *-ed* tokens in a class over the sum of all past tokens) versus frequency (summed over all members) in the final decade. It demonstrates that even a basic phonological classification makes the dependence of regularity on frequency considerably clearer, as contrasted with Fig. 2A.

The insets in Fig. 4 show four representative classes in four snapshots over time. The *hide* and *dwell* classes represent two different states of class instability, while the *sing* and *burn* classes are essentially stable. The *hide* class is destabilized as it actively draws a new irregular (in this case, *light*), but approaches a more stable state towards the end of the time period as *light* fully irregularizes. The *dwell* class, on the other hand, represents a highly destabilized class as its members regularize. Concerning more stable classes, the *sing* class is largely stable in its irregular state, and explains the existence of persistent low frequency irregulars such as *spit*. The *burn* class, on the other hand, is rapidly losing members to regularization, and has almost completely regularized by the end of the time period. Based on these results, the stability of a class seems related to the variance of 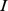 among members of a class (see Figure S6, file S1).

## Discussion

Language is an open system with many different processes acting simultaneously to characterize its dynamics. In our analysis of American English we put a few of these processes in perspective by dissecting the dynamics in verb birth, death, regularization, and irregularization, and estimating their relative relevance. Our study allows for several novel observations about the diachronic dynamics of verb irregularity.

First we observe that, despite the growth of the number of types, the total number of irregular types stays largely constant over time, with a considerable and consistent percentage of tokens (between 65–70%; see Figure S5, file S1) being irregular past tense forms, due to the irregularity and stability of the most frequent verbs. This implies that, despite the increase in expressive power, the cognitive effort required to master the structure of past tense forms is constant over time.

The second important observation comes from the time evolution of the verb system. Important changes occurred during the 160 years considered, with a substantial growth in the percentage of regular verbs. The origin of this variation is exogenous: a large number of new regular verbs are introduced into the language, many more than those (also typically regular) which get extinct. Apart from the large influx of verbs entering the language the vast majority of verbs present over all 16 decades remains in the categories of stable regulars or stable irregulars, with stationary distributions well separated in frequency. However, even in this relatively short time period, we observe some endogenous dynamics in the core vocabulary. Endogenous irregularization, often considered so rare as to be irrelevant, was about as relevant as regularization: the number of regular verbs shifting toward irregular is comparable to the number of irregulars becoming regular. Endogenous dynamics take place within a specific frequency window where transitions in both directions occur, a scenario sharpened by classifying irregular verbs in terms of their phonological similarity.

In conclusion, a diachronic analysis of past tense formation in American English reveals a complex interplay of exogenous and endogenous factors affecting how rules and exceptions coevolve. This points to a more general scenario in which rules in language, far from being fixed over time, emerge, evolve, and compete in a self-organizing way. This self-organization is a response to exogenous pressures exerted by the porous character of the system, as well as the endogenous need to limit cognitive effort while mastering expressivity and comprehension. This work provides a starting point for further studies to investigate the emergence of rules and exceptions in other areas of language, as well as in all the areas of human cognition where norms play a crucial role.

## Materials and Methods

### Corpus preparation

CoHA provides part-of-speech tagged frequency lists covering the period between 1810 and 2010 (over 400 million tokens) using the CLAWS set [34]; we used only the 1830–1989 period (see S3, file S1 for further detail). We began by confining the list from this period only to verbs and lemmatizing it, removing many tagging errors in the process. To avoid potential effects of increasing sample size, we considered an equal number of verb tokens in each decade, according to the size of the first decade considered (1830–1839; 2,177,654 verb tokens). S3 (file S1) provides further detail regarding corpus preparation.

### Proportion of irregularity

The proportion of irregular usage 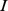 for each verb in each decade was calculated by dividing the number of irregular past tense (non *-ed*) tokens in a decade by the total number of past tense tokens for that verb in the decade. Irregular spellings which do not correspond to irregular pronunciation (e.g., *paid* for *pay*) were considered regular tokens. In some cases, a verb or root could have an undefined 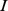 for a given decade, due to extremely low frequency (or non-existent) usage in the past tense. See S4 (file S1) for further details.

### Phonological classes

A full list of all 52 classes and their members is provided in Table S4 (file S1), details about how verb roots were classified are in S5 (both in file S1). Verb roots which exhibited multiple irregular forms were phonologically classified based on their most frequent irregular form (i.e., *swing* was classed with *ring* instead of *string*, although the form *swung* did occur). Suppletive forms such as *do* and *be* were put in their own classes, and forms which did not class identically with any other verbs (e.g. *slay*) were also classed alone.

## Supporting Information

File S1 Contains the following files: **S2**: Glossary of terms used for data and analysis. **S3**: Details of methodology for corpus lemmatisation and preparation. **S4**: Details regarding data analysis. **S5**: Details regarding phonological classes for irregulars. **Figure S1**: Heap and Zipf distributions of verbs in CoHA prior to confining. **Figure S2**: Verb tokens per decade prior to and after confining. **Figure S3**: Frequency histogram of root types by category. **Figure S4**: Temporal trajectory of six transitioning roots. **Figure S5**: Percentage of past tense tokens per decade which are irregular. **Figure S6**: Variance over time of items in irregular phonlogical classes. **Table S1**: Summary of verb token counts per decade prior to and after corpus preparation. **Table S2**: Summary of verb types by category per decade. **Table S3**: Summary of root types by category per decade. **Table S4**: Summary of phonological classes for irregular verbs.
(PDF)

File S2File detailing verb types in each decade with their 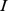 value and adjusted frequency (after confining).(CSV)

File S3File detailing root types in each decade with their 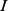 value and adjusted frequency (after confining).(CSV)

## References

[1] Whitney WD (1875) The life and growth of language, an outline of linguistic science. New York: D. Appleton & Company.

[2] Liljencrants J, Lindblom B (1972) Numerical simulation of vowel quality systems: The role of perceptual contrast. Language 48: 839–862.

[3] Lindblom B (1986) Phonetic universals in vowel systems. Experimental Phonology: 13–44.

[4] Labov W (1972) Sociolinguistic patterns. 4. University of Pennsylvania Press.

[5] Mühlhäusler P (1996) Linguistic ecology: language change and linguistic imperialism in the Pacific region. London: Routledge, xiv, 396 p pp.

[6] Mufwene SS (2001) The Ecology of Language Evolution. Cambridge: Cambridge University Press. URL http://groups.lis.illinois.edu/amag/langev/paper/mufwene01book.html.

[7] Lupyan G, Dale R (2010) Language structure is partly determined by social structure. PLoS ONE 5: e8559. 20098492 10.1371/journal.pone.0008559 PMC2798932

[8] McMahon AM (1994) Understanding Language Change. Cambridge University Press, Cambridge UK.

[9] Croft W (2000) Explaining language change: an evolutionary approach. Pearson Education Limited, Essex UK.

[10] Jones MC, Esch E (2002) Language change: the interplay of internal, external, and extralinguistic factors. Walter de Gruter, New York.

[11] Gries ST, Hilpert M (2010) Modeling diachronic change in the third person singular: a multifactorial verb- and author-specific exploratory approach. English Language and Linguistics 14: 293–320.

[12] Hilpert M (2013) Constructional change in English: Developments in allomorphy, word formation, and syntax. Cambridge University Press, Cambridge UK.

[13] Michel JB, Shen YK, Aiden AP, Veres A, Gray MK, et al (2011) Quantitative analysis of culture using millions of digitized books. Science 331: 176–182. 21163965 10.1126/science.1199644 PMC3279742

[14] Perc M (2012) Evolution of the most common english words and phrases over the centuries. Journal of the Royal Society Interface rsif20120491. 10.1098/rsif.2012.0491 PMC3481586 22832364

[15] Petersen AM, Tenenbaum J, Havlin S, Stanley HE (2012) Statistical laws governing fluctuations in word use from word birth to word death. Scientific Reports 2: 1–9. 10.1038/srep00313 PMC3304511 22423321

[16] Petersen AM, Tenenbaum J, Havlin S, Stanley HE, Perc M (2012) Languages cool as they expand: Allometric scaling and the decreasing need for new words. Scientific Reports 2: 1–10. 10.1038/srep00943 PMC3517984 23230508

[17] Pinker S, Ullman M (2002) The past and future of the past tense. Trends in Cognitive Sciences 6: 456–463. 12457895 10.1016/s1364-6613(02)01990-3

[18] Lieberman E, Michel JB, Jackson J, Tang T, Nowak MA (2007) Quantifying the evolutionary dynamics of language. Nature 449: 713–716. 17928859 10.1038/nature06137 PMC2460562

[19] Carrol R, Svare R, Salmons J (2012) Quantifying the evolutionary dynamics of German verbs. Journal of Historical Linguistics 2: 153–172.

[20] Bybee J (2007) Frequency of use and the organization of language. Oxford, UK: Oxford University Press.

[21] Davies M (2012) Corpus of Historical American English: 400 million words from 1810–2010. URL http://corpus.byu.edu.

[22] Pinker S (1999) Words and Rules: The ingredients of language. Harper Collins: New York.

[23] Rumelhart DE, McClelland JL (1986) On learning the past tenses of english verbs. In: McClelland JL, Rumelhart DE, editors, Parallel Distributed Processing (Vol 2): Psychological and Biological Models, Cambridge: MIT Press. pp. 216–271.

[24] McClelland JL, Patterson K (2002) ‘words *or* rules’ cannot exploit the regularity in exceptions. Trends in Cognitive Sciences 6: 464–645. 12457896 10.1016/s1364-6613(02)02012-0

[25] Yang C (2002) Knowledge and learning in natural language. Oxford University Press, Oxford UK.

[26] Heaps HS (1978) Information Retrieval: Computational and Theoretical Aspects. Orlando, FL, USA: Academic Press, Inc.

[27] Zipf GK (1949) Human Behavior and the Principle of Least Effort. Addison-Wesley, Reading MA (USA).

[28] Bybee JL (1985) Morphology: a study of relation between meaning and form. Benjamins: Amsterdam.

[29] Kirby S, Cornish H, Smith K (2008) Cumulative cultural evolution in the laboratory: an experimental approach to the origins of structure in human language. Proceedings of the National Academy of Sciences 105: 10681–10686. 10.1073/pnas.0707835105 PMC2504810 18667697

[30] Albright A, Hayes B (2003) Rules vs. analogy in English past tenses: A computational/experimental study. Cognition 90: 119–161. 14599751 10.1016/s0010-0277(03)00146-x

[31] Pinker S (2000) The irregular verbs. Landfall: 83–85.

[32] Bybee J (2001) Phonology and language use. Cambridge University Press: Cambridge.

[33] Skousen R (1989) Analogical modeling of language. Dordrecht: Kluwer Academic Publishers.

[34] Garside R (1987) The claws word-tagging system. In: Garside R, Leech G, Sampson G, editors, The computational analysis of English: A corpus-based approach, London: Longman. pp. 30–41.

