# Supplementary material for: Internal and External Dynamics in Language: Evidence from Verb Regularity in a Historical Corpus of English
Source: PLoS One. 2014 Aug 1;9(8):e102882. doi: 10.1371/journal.pone.0102882 (PMC4118841; doi:10.1371/journal.pone.0102882)
Supplement: File S1 — Contains the following files: S2: Glossary of terms used for data and analysis. S3: Details of methodology for corpus lemmatisation and preparation. S4: Details regarding data analysis. S5: Details regarding phonological classes for irregulars. Figure S1: Heap and Zipf distributions of verbs in CoHA prior to confining. Figure S2: Verb tokens per decade prior to and after confining. Figure S3: Frequency histogram of root types by category. Figure S4: Temporal trajectory of six transitioning roots. Figure S5: Percentage of past tense tokens per decade which are irregular. Figure S6: Variance over time of items in irregular phonlogical classes. Table S1: Summary of verb token counts per decade prior to and after corpus preparation. Table S2: Summary of verb types by category per decade. Table S3: Summary of root types by category per decade. Table S4: Summary of phonological classes for irregular verbs. (PDF) [file pone.0102882.s001.pdf]

S2: Glossary

- **Token**: A single instance of a verb use in the corpus, e.g., in "I walked, she walks and he talks" there are three verb tokens (*walked, walks, talks*).
- **Type**: All token instances of a given verb lemma, e.g., in "I walked, she walks and he talks", there are two types, *walk* and *talk*. *Walk* has two tokens (*walked, walks*), while talk has one (*talks*). The number of tokens for a given type determines its frequency.
- **Verb Type**: All separate verb lemmas, regardless of shared basic root verb. For example, *do* and *undo* constitute two separate verb types with different token counts.
- **Root Type**: Lemmas collapsed by basic root verb. When referring to root types, tokens of *do* and *undo* both contribute to the frequency of the root type *do*.
- f: The basic usage frequency of a type, calculated as the number of tokens (from all tenses) divided by the total number of tokens in a decade (this number is held constant across decades at the value of 2,177,456 after confining at the size of the first decade, see text).
- f_{past}: The frequency of usage for a lemma in the past tense, defined as the total number of past tense tokens for the lemma divided by the size of the decade (overall number of tokens in decade) prior to confining (shown in Table S1).
- I: The proportion of irregularity for a given type or class, defined as the number of irregular (non *-ed*) past tense tokens for the type divided by the total number of past tense tokens for the type. I can be defined only for types which exhibit a sufficient frequency (see *Undefined*). Each type may have a different I in each decade.
- **Undefined**: A verb or root is considered undefined in a given decade if its $f_{past} < 2.75 \cdot 10^{-6}$. In other words, a type's regularity is undefined if it has extremely low f_{past}.
- **Extended Vocabulary**: Entire set of verb or root types present after confining the corpus size to control for the increase in available text over time. This set includes verbs which enter and/or exit the vocabulary at any point during the 160 year time period.
- **Core Vocabulary**: The set of verb and root types present in all sixteen decades considered. Note that verbs or roots with very low f, though part of the core vocabulary, may have undefined I in some decades.
- **Mostly Regular**: A type is classified as mostly regular if its $I < 0.5$.
- **Mostly Irregular**: A type is classified as mostly irregular if its $I \geq 0.5$.
- ϵ: The error threshold for considering a verb or root to be regular or irregular (see below), set at $\epsilon = 0.01$. In other words, if the I of a type is within ϵ of 0 or 1, it is considered regular or irregular (respectively). Note that a verb which is regular (irregular) in a decade may not stay regular (irregular) in other decades.
- **Stable Regular**: A verb or root is considered a stable regular if it respects $I \geq 0 + \epsilon$ in all 16 decades.
- **Stable Irregular**: A verb or root is considered a stable irregular if it respects $I \geq 1 - \epsilon$ in all 16 decades.
- **Active**: A verb or root is considered active if its I exhibits a value respecting $0 + \epsilon \leq I \leq 1 - \epsilon$ in at least one decade.

S3: Corpus Preparation

This section provides basic information about the corpus, and details regarding our methods in the preliminary stages of preparing the corpus for analysis. The Corpus of Historical American English contains over 400 million words of written English from 1810-2009. Each decade is genre balanced to contain roughly equal representation of fiction and non-fiction sources [2]. CoHA provides tagged frequency lists of words for each decade.

Although CoHA spans 1810-2009, we used only the period between 1830 and 1989. The final two decades were removed (1990-2009) due to the fact that they are duplicated in the larger Corpus of Contemporary American English (CoCA; used for a separate investigation not reported here). The first two decades were removed as they displayed rather extreme growth in the database size. The number of verb tokens in the first decade (1810-1819) is approximately 20% of the size of the second (1820-1829), and the second decade is still only about half the size of the third (1830-1839). Thereafter, growth levels off, with more moderate increases in the number of tokens between decades (see Figure S3, Table S2). We thus discarded the first two decades due to their small relative size; further potential effects of increasing database size were addressed by removing extremely low frequency items and by confining the size of each decade according to the 1830-1839 decade. This is explained in greater detail below.

Lemmatisation, Removals & Confining

Prior to analysis, the corpus was confined only to verbs and hand lemmatised by the authors (i.e., the words *walked*, *walking* and *walks* were all tagged as tokens of the lemma *walk*). In the process of lemmatisation, several types of removals were made. First, all modal auxiliary verbs in all tenses (e.g., *can, could, may, must*) were removed as they are considered function words rather than lexical verbs (and are excluded from earlier studies of regularity, e.g., [5]). Tagging errors, spelling and OCR errors, and items which occurred at extremely low frequency in very few decades were also removed.

Hand lemmatisation allowed the coders to check many obvious tagging errors against their context in the corpus and remove such errors altogether. For example, *chung* is tagged as a past tense verb, but invariably occurs as a proper noun (e.g., in a context such as "I told her very briefly Chung Bong's story" [2]). Note that not all tagging errors were removed; only those which were unknown English words and which had low enough frequency to allow checking against the corpus itself to verify the error[1]. Where a past tense verb had the potential for error (e.g., *abandoned* can be either an adjective or verb, and adjective tokens were sometimes incorrectly tagged as verbs as in "this dear abandoned innocent" [2]), but this error was not uniform (i.e., most tokens of *abandoned* tagged as the past tense were correctly tagged), all tokens were included. This is due to the fact that manually checking all contexts of a particular word form is infeasible in CoHA (due to copyright restrictions at the time of analysis) for words which do not have a low token count.

Spelling or optical character recognition (OCR) errors were corrected where possible, for example *abandonedthe* was coded as a past tense token of *abandon*, and *aasserting* is a clear instance of a misspelling of *asserting*. Where these types of errors could not be straightforwardly interpreted, (e.g., for stranded bound morphems like *-ify*, where each occurence was an error of the end of a different word, such as *qual-ify, sign-ify*), they were removed entirely. Verbs were also removed if they did not occur with a frequency of more than 10^{-8} in at least three decades. In other words, as our analysis aimed to observe changes over time, we discarded verbs with both extremely low frequency *and* a very short lifespan (<30 years total, though not necessarily consecutive) in the corpus.

These three criteria for removal led to the removal of between $9 - 12\%$ of the verb tokens in each decade (see Table S2). Figure S3 contrasts the token count of all verbs in the corpus (Davies, personal communication) versus the token count after our removals from each decade. The removal of modals accounted for the loss of between $6 - 8\%$ of tokens (between $55 - 80\%$ of all removals), depending on the decade. This percentage drops over time, indicating that the proportion of modals as a proportion of all verbs drops over time. This is likely due to the growth in the number of new lexical verbs evident even with the corpus size constrained (see main text, Figure 1). Additionally, the percentage of removal due to other criteria also increases over time (see Table S2); this is due to an increase in tokens removed due extremely low frequency items which occur for very short periods of time. This is consistent with an increase in the database size (more tokens can lead to the introduction of more low frequency types [3]), rather than any major variations in tagging errors, estimated to be stable around $1 - 2\%$ overall [2]. Moreover, the direction of these removals is conservative with respect to our results; in other words, even though a larger proportion of verb tokens were removed from the later decades, we still observed a growth in the number of verbs over time (see main text).

[1] Although it is worth noting that tagging errors are more likely for low frequency items amenable to verification [2].

An increase in database size has the potential to affect the number of types observed [3]. Despite having removed very briefly or sporadically occurring low frequency items in the coding process, the growth in the number of tokens over time is still considerable (see Figure S3). To consider genuine vocabulary growth (rather than the growth in text available for digitized corpora over time), we confined the number of verbs considered in each decade to the number of tokens in the smallest decade (1830-1839; 2,177,456 tokens). This involved recreating a random sequence of all observed verb tokens in each decade. We then drew 2,177,456 tokens from each randomly sequenced set of verbs (with the exception of the first decade considered, 1830-1839, which remained intact). Verb types which remained after the set was confined constitute the extended vocabulary considered in our final analysis. Frequencies (f) were calculated based on the number of tokens per lemma divided by the size of the confined set.

S4: Data Preparation

After confining the size of each decade, we analysed several fundamental properties of the data: entrances and exits, frequency, root types, and regularity. Entrances and exits were considered in terms of single decade interval, meaning *every* entrance and exit was counted, even if the same verb entered and then exited in consecutive decades. In other words, the verbs entering between 1840 and 1850 are verbs appearing in 1850 which did not appear in 1840, regardless of whether they appeared in 1830. However, overall, more verbs enter and stay (or enter more times than they exited), making the net result a growth in the number of verbs (see Figure 1E, main text).

Regularity

Types in the extended vocabulary were categorized according to their proportion of irregularity (I), *i.e.*, the fraction of irregular simple past tense occurrences over the total number of past tense tokens. The I was calculated from our lemmatized version of the verb set, prior to confining decade size. Since the purpose of confining was to control for frequency effects related to database size, the process of confining did not recreate a *tagged* database of verb tokens; information regarding past tense regularity is only available from the original lemmatized version. The I was calculated using only the simple past tense; irregularity in the past participle was not considered, such that e.g., *prove* is entirely regular (e.g., *I proved her wrong*) although it has an irregular past participle (e.g., *It has proven difficult*) in common usage. Irregular spellings such as *paid* for the past tense of *pay* (as opposed to *payed*, which also occurs in the corpus) were considered regular past tense tokens, since spelling irregularity and variation were not considered in our analysis. Figure S5 shows the proportion of irregular tokens overall, which indicates that between $65-70\%$ of all past tense utterances are irregular. Even with the removal of the highest frequency irregulars, *be* and *have* (which also have the potential to be function verbs), around 50% of all past tense verb tokens are irregular. This indicates that while regularity dominates types, irregularity dominates tokens.

We considered regularity undefined if past tense usage was so infrequent (or non-existent) that the regularity of a verb could not be determined without the potential for error. Therefore, in order to have defined regularity, a verb had to have past tense usage greater than or equivalent to a frequency of $2.75 \cdot 10^{-6}$ in a given decade. Because past tense usage and irregularity is based on the unconfined corpus, past tense usage frequency was calculated according to the original lemmatized corpus size for each decade. This frequency threshold is equivalent to at least 6 past tense tokens for the first decade (1830-1839), but the number of past tense tokens required to reach this threshold scales with the increase in corpus size (such that e.g., at least 14 past tense tokens are required to pass the threshold in the final decade). Frequency of usage in the past tense scales with overall frequency, such that low frequency items are much more likely to be undefined.

For broad contrasts in the extended vocabulary, all verbs were classified as mostly regular or mostly irregular ($I < 0.5$ and $I \geq 0.5$, respectively). However, the remainder of the analysis leveraged the availability of a scalar I by contrasting regular and irregular roots with active roots in the core vocabulary. Root types with an $I \leq 0 + \epsilon$ were labelled as regulars, root types with $I \geq 1 - \epsilon$ irregulars. When considering decades separately, active types are only considered active in decades where their $I \geq 0 + \epsilon$ or $I \leq 1 - \epsilon$, but are considered (ir)regular elsewhere. However, across the entire time period, stable regulars or irregulars are types with an $I \leq 0 + \epsilon$ or $I \geq 1 - \epsilon$ in every decade. Consequently, types with a $I \geq 0 + \epsilon$ or $I \leq 1 - \epsilon$ in at least one decade were labeled as active across the time period.

The extended vocabulary presented with 6885 unique verb types. In order to examine the contribution of genuinely new verbs, exclusive of the contribution of new verbs which used an existing verb root productively, verbs were collapsed by their roots. This was particularly important since the use of existing irregular verb roots contributed in part to the introduction of "new" irregular types in the period. To this end, each of the 6885 verb types was assigned a root. The vast majority of verbs were monomorphemic and thus identical to their roots; e.g., the verb *usher* is identical to the root *usher* in all decades. Even many multimorphemic verbs were also identical to their roots, since words were only classed by free *verb* roots, as irregularity can only be "inherited" from a verb root [7]. For example, the verbs *slave* and *enslave* constitute separate verbs as well as separate roots, since they derive from the noun *slave*, and thus do not share a verb root. Collapsing verbs by their roots resulted in a reduction in the number of unique types in the extended vocabulary, to 5791. Tables S3 and S4 summarize types by decade in terms of verbs and roots, respectively. The values of f and I for roots were re-calculated with the number of root tokens over decade size (for f) and the total number of irregular past tense tokens over the total number of past tense tokens for the entire root (I)[2].

[2]This makes it possible that in the case of the number of active types in a single decade, there are more active roots than active verbs. This is because using multiple verbs to create a single I drives some additional roots into transition.

Of 200 unique irregular verb types (151 root types) in the corpus, 18 (9%) of these appear after 1840, while 22 (11%) are lost, making for a small net decrease in the number of irregular verb types (the remaining 80% are in the core vocabulary). In the case of regulars, the birth rate observed is not only much greater than that of irregulars, but it dwarfs the death rate; 26.3% of all regular verb types in the corpus are born after 1840, while only 14.3% of verbs are lost.

Calculation of root types shows that the 18 entrances of irregular verbs occur either because of definition or root proliferation (i.e., the productivity of an existing irregular root, as in *do-undo*). Definition occurs when a verb acquires sufficient frequency of usage in the past tense for its regularity to be reliably defined; i.e., it moves out of the undefined category. Four of the 18 entering irregulars (approximately 23%) are undefined in the early decades, but enter as mostly irregular ($I \geq 0.5$) at their first occurrence and remain mostly irregular throughout their lifetime. The largest percentage of irregular verb birth is accounted for by the proliferation of irregular roots; 11 of the 18 nascent irregulars (just over 60%) are multi-morphemic verbs using an existing irregular root, such as *outdo* and *override*. The remaining three new irregular verbs (constituting 17%), are not entrances, rather, they are instances of irregularization: verbs which at their first occurrence were regular, but by the final decade have become irregular. If verbs are collapsed by their roots - eliminating the process of root proliferation as a mechanism of birth, this leaves only 7 new irregular roots (of 151 unique root types total): 3 irregularizations and 4 definitions.

Unlike for irregulars, root proliferation is not a major contributing force behind new regular types, since collapsing regular types into roots has little effect on the observed rates of birth and death (adjusting them to 26% and 13.8%, respectively). Definition accounts for a large proportion of new regular verb types, with 78.7% of new regulars becoming defined as regular sometime after 1840 (although they occur with some f in early decades). Verbs entering the system form the second largest source of new regulars, accounting for almost 21% of new regulars. Lastly, regularization accounts for a small minority of new regulars, with only three verbs regularizing completely, constituting less than 0.5% of regular verb growth. In other words, defining and entering verbs skew drastically towards being regular, and a growth of the number of types over time is the primary force driving an overall increase in regularity in the language system.

S5: Phonological classes

All verbs which had a non-zero I at any time during the 160 years examined were classed according to the change from the infinitive form to the irregular past tense form. A full list of all 52 classes and their members is provided in Table S5. Verbs which exhibited multiple irregular forms were phonologically classified based on their most frequent irregular form (i.e., *swing* was classed with *ring* instead of *string*, although the form *swung* did occur in a minority). Suppletive forms such as *go* and *be* were in their own class, and forms such as *slay/slew* and *lay/lay* did not class identically with any other verbs, and were thus also classed alone. Each class has an f defined as the sum of the frequencies of its root members, while the I in each class is calculated by dividing the sum of irregular past tense tokens in the class by the sum of all past tense tokens.

These classes may not be optimal, and could in fact be more or less fine grained. For example, this metric is coarse in that it does not take into account the presence of several complex onsets in irregulars (e.g., *spr-, str-*), which may effect irregularisation (in other words, it does not use overall phonological distance; see [1]). On the other hand, most systems of classification are much broader with as few as 6-8 classes encompassing all irregular verbs [4, 6].

Because an irregular form is required for class membership, class sizes are not fixed over time. In other words, while irregulars have the potential to contribute to classes, regulars do not (such that, e.g., there is no subset of regular classes with phonologically similar members). When a particular root type regularizes completely, it leaves its irregular class entirely; in other words, the class can be said to be losing a member to regularization. For example, the verb *work* occurs early on in the corpus with an I of 0.04 as there is still some usage of the irregular form *wrought*. While *work* has this positive I, it belongs to the *teach* class. However, by 1930, *work* has an I of 0, and has therefore left the *teach* class. Thus, in 1930, the *teach* class shrinks in overall size, and *work* no longer contributes to either the class's f or its I. Likewise, each class also has the potential to gain members as new irregular forms emerge (or new verbs enter as irregular, although this is rare in our data). For example, the verb *ruin* occurs only with the form *ruined* until 1880, at which point the form *ruint* emerges. As *ruin* now has an I of 0.02, it enters to the *burn* class contributing to both its f and I.

Figure S1: Heaps curves (A) and Zipfian distributions (B) prior to confining the dataset to the size of the 1830-1839 decade.

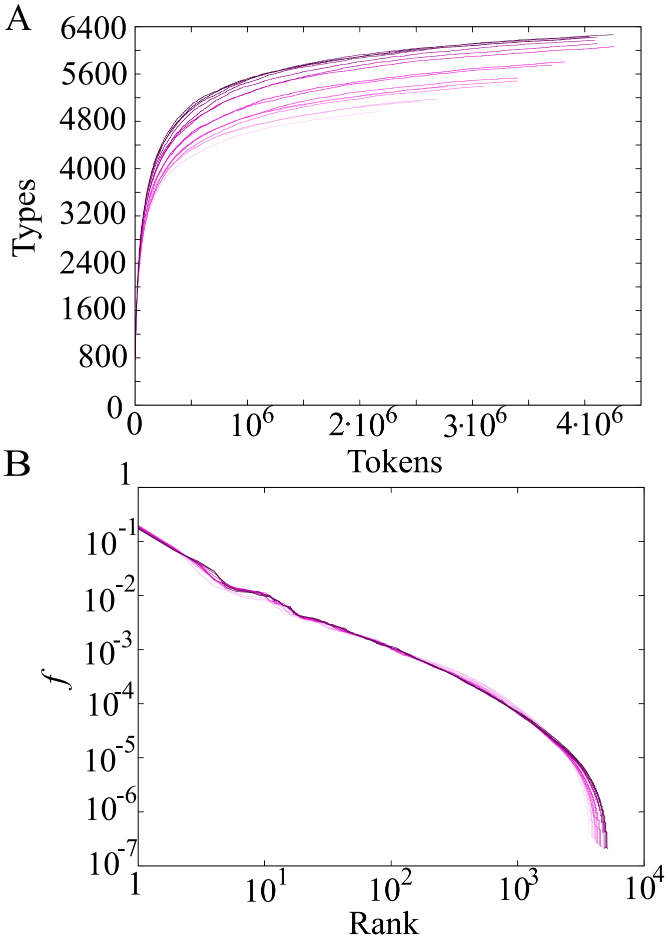

Figure S2. Number of verb tokens in CoHA in each decade. The red line indicates the number of all tokens prior to removal of modal auxiliaries, obvious errors, and extremely low frequency/sporadically occurring types, while the blue line indicates the number of tokens after this removal.

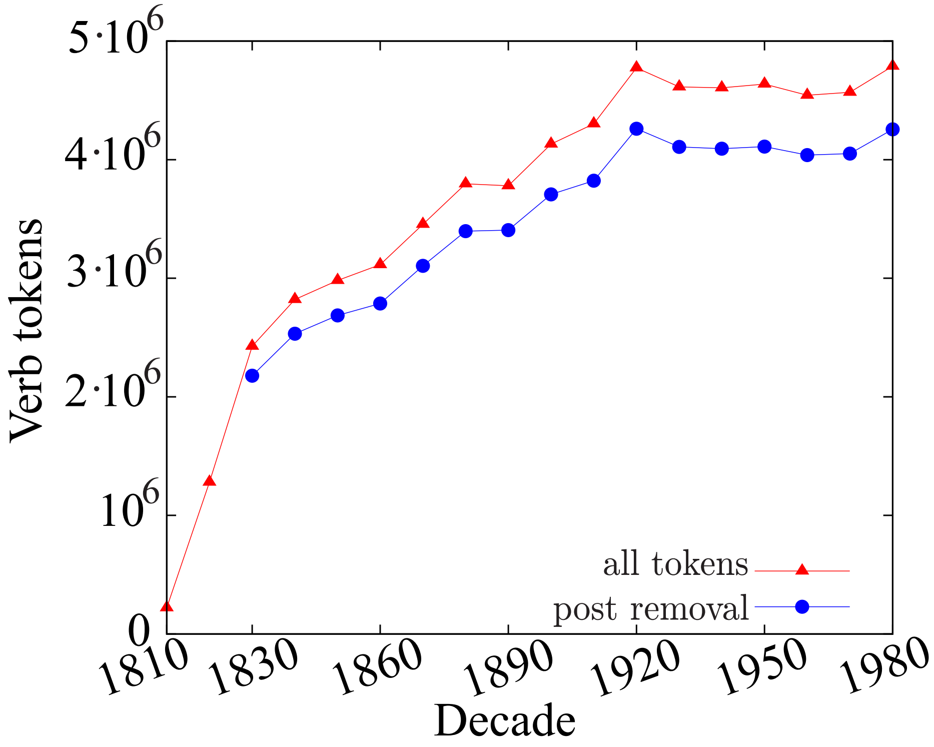

Figure S3. Depicts the frequency histogram of root types divided by category (regular, irregular, undefined and active) in four different decades (compare to Figure 1E in the main text, which depicts mostly regular and mostly irregular root types). This shows that the growth in number of types is mainly a consequence of entering regular types, many of which were simply previously undefined. The starting point of the first frequency bin for each decade is indicated.

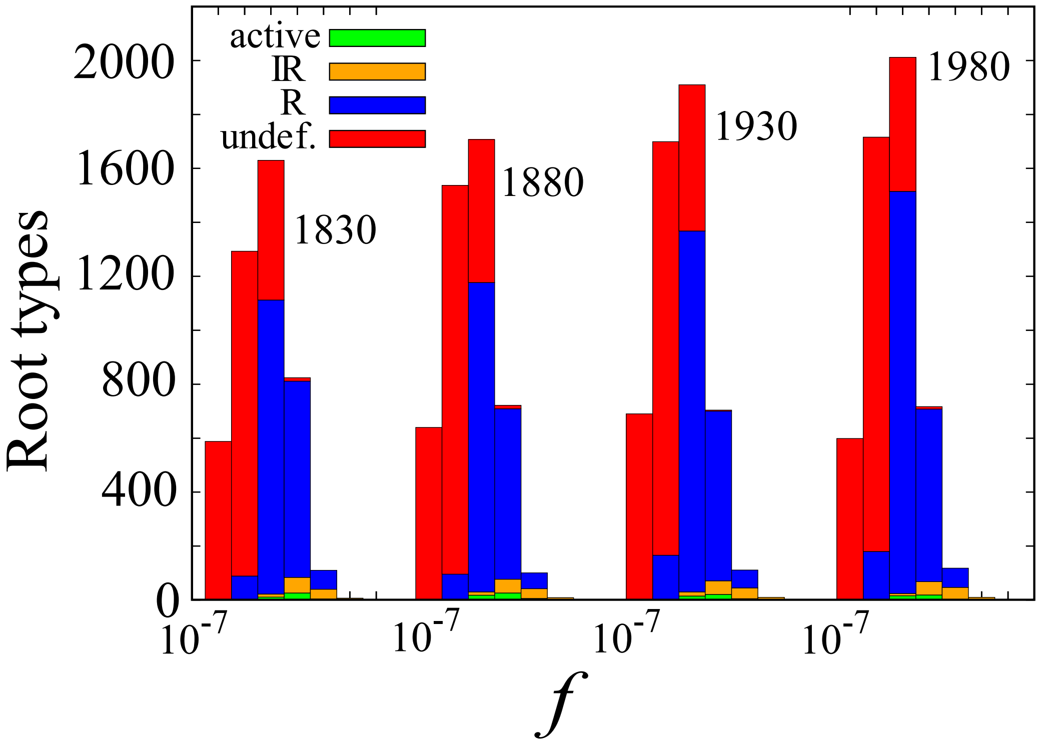

Figure S4. Transitioning roots. The six roots in the database which transition from (A) mostly irregular to mostly regular, and (B) mostly regular to mostly irregular.

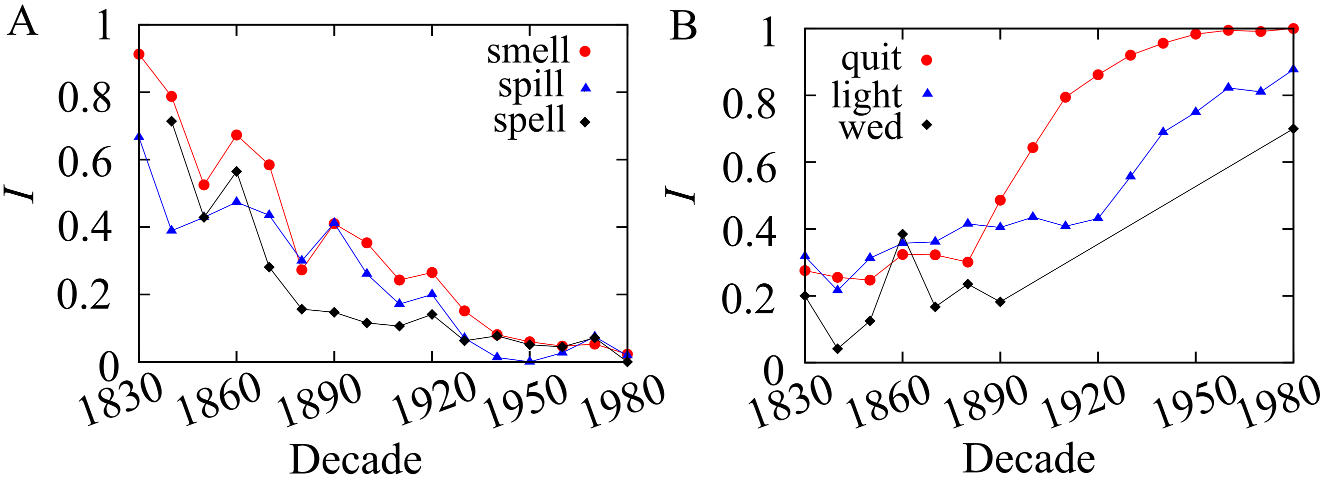

Figure S5. Proportion of irregular past tense tokens, I_{tot}, in each decade for all verbs (red line) and excluding excluding particularly high frequency types “be” and “have” (blue line).

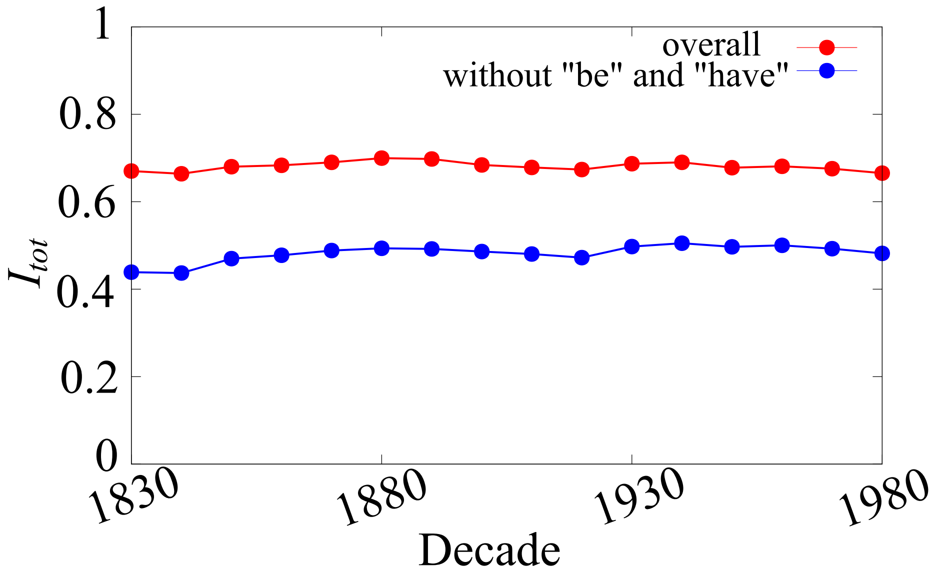

Figure S6. Plot of I versus the f_{sum} for each phonological class over time. Time is represented by color (red hue for the first decade, blue hue for the last decade). Each member of a class is plotted individually (with its own I and f) and connected to the class by a line. The size of the circle depicting the class indicates how many members are in the class.

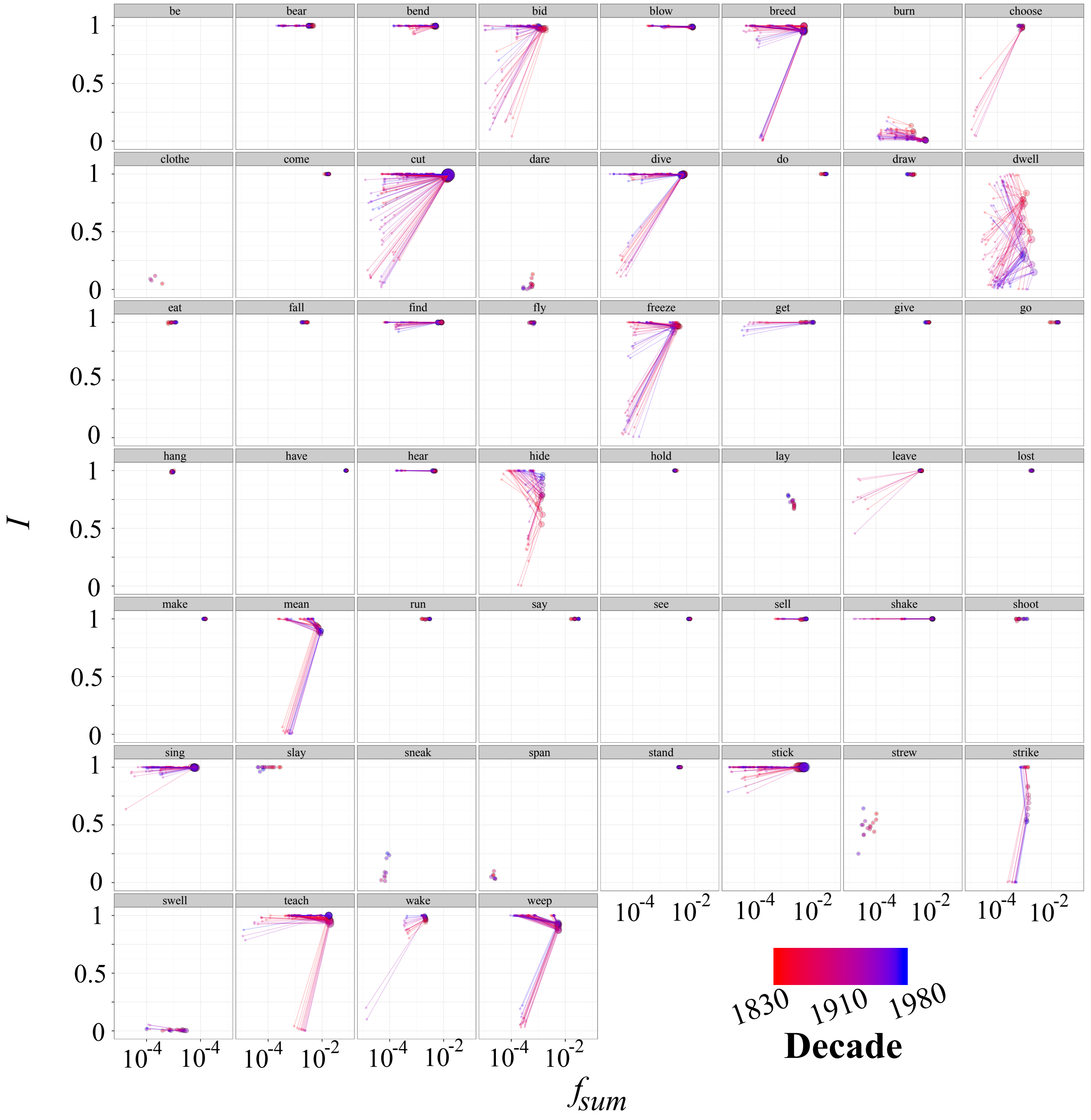

Figure S7. Visualization of the variance in regularity among classes. In each decade, for a given class with a size, s, we can define its variance, σ, as:

$$\sigma = \frac{\sqrt{\sum_v (I_r - I_c)^2}}{\sqrt{s}},$$

Where I_r is the proportion of irregularity of each member root of the class, and I_c is the overall I of the class. This figure shows a plot of the variance of σ over time against the average σ ($\bar{\sigma}$) for each class. Classes with higher $\bar{\sigma}$ are in the process of losing a member throughout the time period, while higher variance in σ indicates the loss of a member. For example, the *mean* class is slowly losing *dream* and *lean*, which have an I of 0.108 and 0.007 respectively by the final decade. The *choose* and *strike* classes both lose members (*behoove* and *climb*, respectively) in the time period. Classes with low variance in σ over time and low $\bar{\sigma}$ are highly stable (and/or have only a single member, e.g., the *be* class).The variance of σ over time versus $\bar{\sigma}$ for all classes. Only classes with a variance over time in σ>0.05 are labelled.

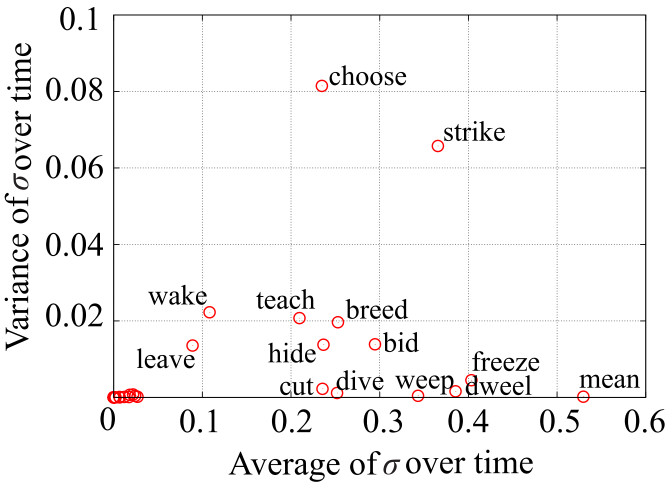

Table S1. **Summary of verb tokens removed from the original, unconfined CoHA set.** Percentages show removals as a percentage the original number of tokens.

Decade	All Verb Tokens	Post Removals	Removed	Modals Removed	Other Removals
1830-1839	2426234	2177456	10.25%	8.34%	1.91%
1840-1849	2820505	2531984	10.23%	8.29%	1.94%
1850-1859	2981280	2686698	9.88%	8.35%	1.54%
1860-1869	3114518	2787135	10.51%	8.14%	2.38%
1870-1879	3454665	3104851	10.13%	8.11%	2.02%
1880-1889	3795539	3397440	10.49%	8.11%	2.38%
1890-1899	3780288	3405447	9.92%	7.65%	2.27%
1900-1909	4131287	3706878	10.27%	7.49%	2.78%
1910-1919	4301527	3823067	11.12%	7.38%	3.74%
1920-1929	4773647	4260603	10.75%	7.04%	3.71%
1930-1939	4612946	4107362	10.96%	6.93%	4.03%
1940-1949	4605199	4092289	11.14%	6.86%	4.28%
1950-1959	4636677	4110181	11.36%	6.83%	4.53%
1960-1969	4543216	4037815	11.12%	6.77%	4.36%
1970-1979	4567916	4050662	11.32%	6.61%	4.72%
1980-1989	4787625	4255111	11.12%	6.33%	4.79%

Table S2. **Summary of verb type categories across decades**

Decade	Mostly Irregular	Mostly Regular	Irreg-ular	Regular	Active	Entering In	Exiting After	Undefined
1830-1839	172	2061	148	2045	40	—	295	2731
1840-1849	172	2149	149	2130	42	389	335	2737
1850-1859	171	2080	152	2064	35	355	256	2827
1860-1869	174	2181	147	2161	47	473	382	2940
1870-1879	172	2126	150	2107	41	319	337	2934
1880-1889	171	2038	144	2012	53	373	346	3059
1890-1899	175	2096	153	2072	46	400	319	3051
1900-1909	173	2144	150	2122	45	504	357	3190
1910-1919	170	2189	147	2168	44	395	267	3186
1920-1929	171	2332	147	2311	45	499	355	3274
1930-1939	172	2290	150	2271	41	391	336	3351
1940-1949	169	2327	146	2306	44	380	359	3361
1950-1959	168	2347	144	2332	39	407	356	3390
1960-1969	169	2314	150	2296	37	399	355	3465
1970-1979	171	2334	149	2315	41	345	336	3433
1980-1989	168	2475	146	2461	36	356	—	3315

Table S3. **Summary of root type categories across decades**

Decade	Mostly Irregular	Mostly Regular	Irreg-ular	Regular	Active	Entering In	Exiting After	Undefined
1830-1839	138	1992	112	1976	42	—	243	2323
1840-1849	138	2071	112	2053	44	308	274	2309
1850-1859	136	2012	117	1996	35	281	187	2377
1860-1869	137	2109	112	2088	46	391	303	2483
1870-1879	135	2048	115	2027	41	240	252	2483
1880-1889	134	1959	109	1933	51	303	287	2624
1890-1899	134	2026	113	2002	45	297	235	2567
1900-1909	137	2078	116	2056	43	405	272	2682
1910-1919	135	2126	113	2104	44	284	175	2648
1920-1929	136	2257	113	2235	45	366	250	2707
1930-1939	137	2219	113	2199	42	275	244	2769
1940-1949	135	2253	112	2232	44	250	246	2743
1950-1959	136	2279	113	2263	39	284	249	2754
1960-1969	136	2242	117	2223	38	270	246	2812
1970-1979	137	2267	116	2247	41	233	242	2773
1980-1989	135	2397	113	2382	37	238	—	2641

Table S4. **Summary of the phonological classes implemented**

Root Members	Change
**be**	be → went (suppletive)
**bear**, swear, tear, wear	/ɛ/ → /o/
**bend**, build, lend, send, spend	/d/ → /t/
**bid**, braid, rid, shed, sled, spread, wed	/d/ → /d/ +∅
**blow**, grow, know, throw	/oʊ/ → /u/
**breed**, bleed, feed, lead, meet, plead, read, speed	/i/ → /ɛ/
**burn**, drown, learn, ruin	/n/ → /nt/
**choose**, behoove	/u/ → /oʊ/
**clothe**	/oʊð/ → /æd/
**come**	/ʌ/ → /æ/
**cut**, beat, bet, burst, bust, cast, cost, hit, hurt, knit, let, put, quit, set, shut, slit, split, thrust, wet	/t/ → /t/ +∅
**dare**	dare → durst (suppletive)
**dive**, drive, ride, rise, shine, smite, stride, strive, write	/aɪ/ → /oʊ/
**do**	do → did (suppletive)
**draw**	/aə/ → /u/
**dwell**, heal, kneel, scare, smell, spill, spell, spoil	alveolar approximant + t
**eat**	/i/ → /æ/
**fall**	/aə/ → /ɛ/
**find**, bind, grind, wind	/aɪ/ → /aʊ/
**fly**	/aɪ/ → /u/
**freeze**, heave, speak, squeeze, steal, weave	/i/ → /oʊ/
**get**, tread	/e/ → /ɑ/
**give**	/ɪ/ → /eɪ/

Root Members	Change
**go**	go → went (suppletive)
**hang**	/æ/ → /ʌ/
**have**	/v/ → /d/
**hear**, flee	/i/ → /e/ + word final /d/
**hide**, bite, light, slide	/aɪ/ → /ɪ/
**hold**	/o/ → /e/
**lay**	/ɛɪ/ → /ɛɪ/
**leave**, cleave	/iv/ → /ɛft/
**lose**	/u/ → /ɑ/ + word final /t/
**make**	/k/ → /d/
**mean**, deal, dream, feel, lean	/i/ → /e/ + word final /t/
**run**	/ʌ/ → /æ/
**say**	/ɛɪ/ → /ed/
**see**	/i/ → /aə/
**sell**, tell	/ɛ/ → /o/ + word final /d/
**shake**, forsake, take	/ɛɪ/ → /œ/
**shoot**	/u/ → /ɑ/
**sing**, drink, ring, shrink, sink, sit, spit, spring, stink, swim	/ɪ/ → /æ/
**slay**	/eɪ/ → /u/
**sneak**	/i/ → /ʌ/
**span**	/n/ → /n/
**stand**	/æ/ → /ʊ/
**stick**, begin, cling, dig, fling, sling, slink, spin, sting, string, swing, win, wring	/ɪ/ → /ʌ/
**strew**	/u/ → /u/
**strike**, climb	/aɪ → /ʌ/
**swell**, help, step	/e/ → /o/
**teach**, beseech, bring, buy, catch, fight, seek, think, work	Replace final syllable rime with /aət/ (Ruckumlaut)
**wake**, break	/ɛɪ/ → /oʊ/
**weep**, creep, keep, leap, sleep, sweep	/i/ → /ɛ/ + word final /t/

References

[1] A. Albright and B. Hayes. Rules vs. analogy in English past tenses: A computational/experimental study. *Cognition*, 90:119–161, 2003.

[2] M. Davies. *Corpus of Historical American English: 400 million words from 1810-2010*. 2012.

[3] Martin Gerlach and Eduardo G. Altmann. Stochastic model for the vocabulary growth in natural languages. *Phys. Rev. X*, 3:021006, May 2013.

[4] S. Greenbaum and R. Quirk. *A student's grammar of the English language*. Longman, London, 1996.

[5] E. Lieberman, J.-B. Michel, J. Jackson, T. Tang, and M. A. Nowak. Quantifying the evolutionary dynamics of language. *Nature*, 449(2007), 2007.

[6] J. L. McClelland and K. Patterson. 'words *or* rules' cannot exploit the regularity in exceptions. *Trends in Cognitive Sciences*, 6(11):464–645, 2002.

[7] S. Pinker and A. Prince. Regular and irregular morphology and the psychological status of rules of grammar. In S. D. Lima and R. L., editors, *The reality of linguistic rules*, pages 321–352. John Benjamins, Philadelphia, 1994.
